# Concurrent adrenal and extra-adrenal myelolipoma: A case report

**DOI:** 10.1016/j.ijscr.2024.109398

**Published:** 2024-02-26

**Authors:** Golnaz Moradi, Diana Zarei, Mahbod Issaiy

**Affiliations:** a Department of Radiology, Sina Hospital, Tehran University of Medical Sciences, Tehran, Iran; b Advanced Diagnostic and Interventional Radiology Research Center (ADIR), Tehran University of Medical Sciences, Tehran, Iran

**Keywords:** Myelolipoma, Retroperitoneal, Adrenal, Computed tomography

## Abstract

**Introduction:**

Myelolipoma, a benign tumor characterized by mature fat cells and hematopoietic cells, is predominantly found in the adrenal glands, accounting for 6–16 % of all adrenal tumors. These tumors are often asymptomatic and discovered incidentally during imaging. We present a rare case of concurrent adrenal and extra-adrenal myelolipomas, contributing to the limited research in this area.

**Case presentation:**

A 65-year-old female with a history of Steven-Johnson syndrome presented with epigastric pain, initially diagnosed with emphysematous cholecystitis. Imaging revealed unexpected lesions near the left kidney. During surgery for presumed cholecystitis, significant hemorrhaging occurred following an attempted biopsy of the left adrenal lesion. This complication necessitated a complete adrenalectomy. Pathological examination confirmed the presence of myelolipomas in the left adrenal gland, para-aortic, and left para-iliac regions.

**Discussion:**

The simultaneous occurrence of adrenal and extra-adrenal myelolipomas is exceptionally rare, posing diagnostic and management challenges. This case highlights the complexity of managing patients with multiple comorbidities and the critical importance of differentiating myelolipomas from other fat-containing retroperitoneal masses. The incidental discovery of these tumors and their potential for significant intraoperative complications, as seen in our case, underscores the need for careful surgical planning and thorough preoperative assessment.

**Conclusion:**

This case emphasizes the diagnostic challenges and management complexities in patients with incidental findings of myelolipoma, particularly when accompanied by significant medical histories. The occurrence of unexpected intraoperative complications highlights the importance of cautious decision-making in surgical interventions. This report provides valuable insights into the unpredictable nature of medical practice and the management of rare pathologies.

## Highlights

• This case report details the uncommon coexistence of adrenal and extra-adrenal myelolipomas, highlighting their diagnostic challenges and importance for medical knowledge.
• Emergency adrenalectomy due to severe hemorrhage during biopsy underscores the complexities and risks in treating myelolipomas.
• The case provides insights into surgical decisions, emphasizing the need for thorough preoperative assessments and flexibility in managing unexpected intraoperative issues.

## 1 Introduction

Myelolipoma, a benign tumor characterized by its composition of mature fat cells and blood-forming (hematopoietic) cells, stands out as a notable abnormality in adrenal gland disorders [1]. It ranks as the second most frequent benign tumor in the adrenal glands, accounting for about 6 to 16 % of all adrenal tumors [2]. Although these tumors are primarily found in the adrenal glands, they can sometimes occur in other areas outside the peritoneum, the most common of which is the presacral region [3]. Typically affecting adults between 55 and 65, myelolipomas show no preference for gender, affecting males and females equally [4,5]. Intriguingly, about 90 % of these tumors are not symptomatic and are discovered only incidentally during imaging procedures intended for other health issues [4,6,7].

In imaging studies, myelolipomas display diverse appearances: they exhibit mixed echogenicity on ultrasound, show prominent fatty areas mixed with denser regions on CT scans, and appear bright in fatty areas on T1-weighted MRI images and moderately bright on T2-weighted images. CT scans are favored for diagnosis, especially when hemorrhage alters the tumor's appearance [3].

The simultaneous occurrence of adrenal and extra-adrenal myelolipomas is highly uncommon, presenting distinctive challenges in their diagnosis and treatment. Our case report which is reported in line with SCARE criteria [8] contributes significantly to the sparse research in this area, documenting a rare instance of a myelolipoma in both the adrenal gland and retroperitoneal area, and aims to improve the understanding and management of such complex cases.

## 2 Presentation of case

A 65-year-old female, with a prior diagnosis of Steven-Johnson syndrome, was admitted to the internal medicine department presenting with dermatological symptoms. She reported experiencing epigastric pain, primarily located in the right upper quadrant. Her clinical assessment, which included raised liver function test results and sonographic findings, suggested an initial diagnosis of cholecystitis. As a result, she was transferred to our facility for more specialized care. The patient's medical background is notable for conditions such as major depressive disorder, hypothyroidism, and Steven-Johnson syndrome. Her surgical history includes procedures for umbilical hernia repair. Her treatment regimen comprises various medications, including metoprolol, valproic acid (Depakine), lithium, levothyroxine, biperiden, gabapentin, aripiprazole, and clonazepam.

During the initial examination upon admission, the patient exhibited tenderness in the epigastric region and the right upper quadrant of the abdomen. Mild tenderness was noted in other abdominal regions, but there were no signs of rebound tenderness or abdominal guarding. Laboratory tests revealed leukocytosis, with a white blood cell count recorded at 25.7 × 10^3^/μL. Liver function tests showed abnormal elevations: aspartate aminotransferase (AST) levels at 77 U/L, alanine aminotransferase (ALT) at 80 U/L, and alkaline phosphatase (ALP) at 525 U/L. Furthermore, the patient's C-reactive protein (CRP) level was significantly increased, measuring at 95.5 ng/L.

Following the patient's admission, another ultrasound examination was performed, revealing characteristics aligned with emphysematous cholecystitis. The ultrasound showed increased thickness and layering in the gallbladder wall, containing some echogenic foci. Importantly, the Murphy sign was absent. Additionally, two hyperechoic lesions were incidentally identified: a larger lesion measuring approximately 96 × 88 mm near the upper pole, and a smaller one, about 45 × 37 mm, near the lower pole of the left kidney. Both lesions were not structurally connected to the kidney. For further assessment, a CT scan of the abdomen with intravenous contrast was advised by the radiologist.

**Fig. 1:**
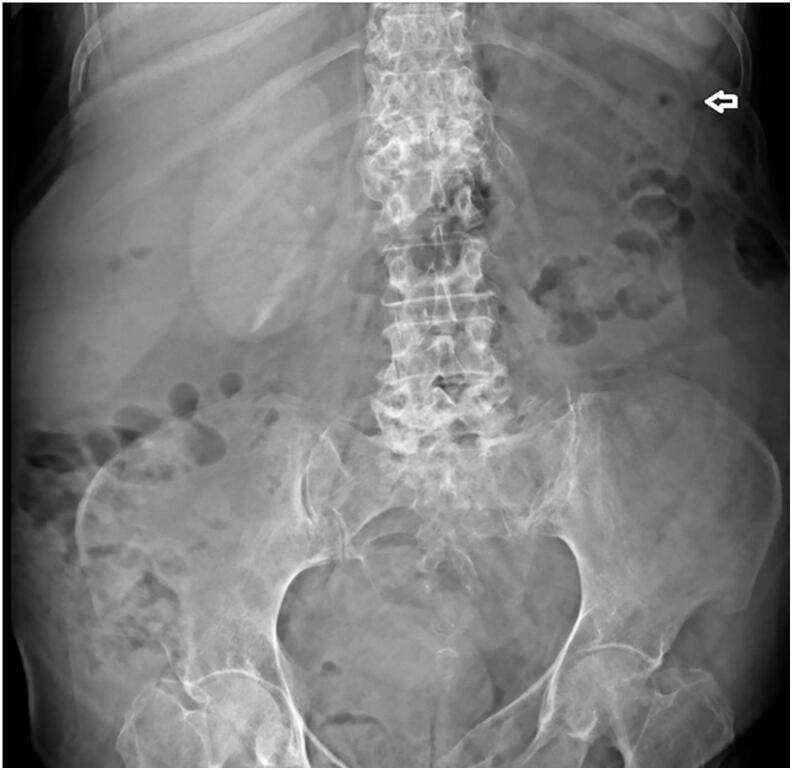
Plain abdominal radiograph (anteroposterior): pressure effect on and downward displacement of left kidney by a well- defined low-density mass is seen.

**Fig. 2:**
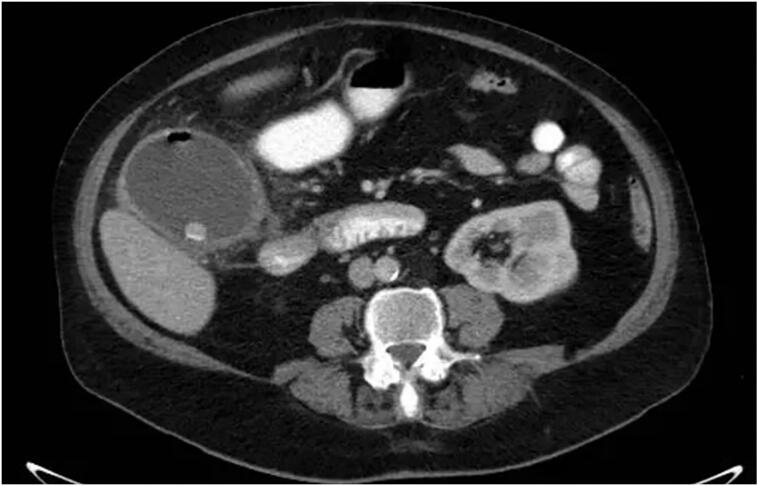
Axial CECT (contrast-enhanced computed tomography) of the abdomen: Enlargement and thickening of the gallbladder wall are evident. The presence of numerous stones, along with a minor quantity of air within the wall and lumen, and pericholecystic fat stranding were observed. These observations align with a diagnosis of emphysematous cholecystitis.

**Fig. 3:**
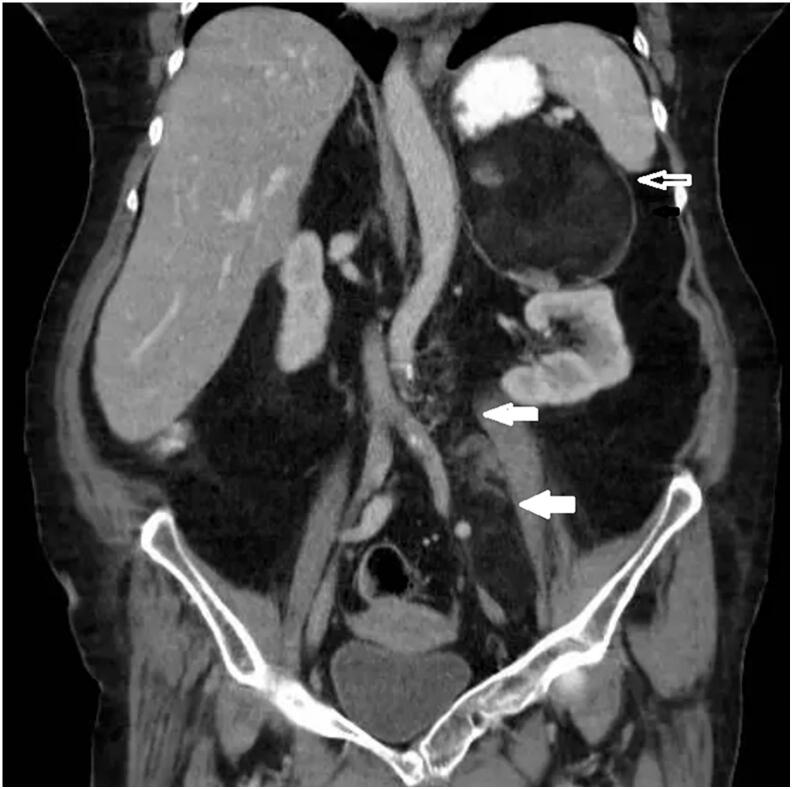
Coronal CECT (contrast-enhanced computed tomography) of the abdomen: the image reveals a round left adrenal mass with primarily fatty composition and soft tissue elements, indicative of myelolipoma (hollow arrow). Additionally, a lesion exhibiting fat density with soft tissue elements is observed in the para-aortic area, extending downward into the pelvic cavity near the left iliac vessels, raising suspicion for liposarcoma (solid arrows).

Fig. 1 features an anteroposterior abdominal X-ray showing a low-density mass applying pressure to and displacing the left kidney downwards. Further confirmation comes from the contrast-enhanced CT scan (CECT) which not only corroborates these findings but also identifies emphysematous cholecystitis (Fig. 2). This scan also highlights a mass in the retroperitoneal area near the left kidney, indicative of a possible liposarcoma. Fig. 3 provides a detailed view of a left adrenal mass, predominantly fatty with interspersed soft tissue, aligning with the characteristics of a myelolipoma. Additionally, a lesion in the para-aortic area is seen, comprising fat and soft tissue and extending towards the pelvic cavity near the left iliac vessels, again suggesting the presence of a liposarcoma.

The patient was prepared for an open laparotomy. During the surgery, conducted via a midline incision, a markedly enlarged, emphysematous gallbladder was discovered, necessitating cholecystectomy. An attempt to biopsy an adrenal tumor led to significant hemorrhaging at the adrenal site, which could not be effectively managed. As a result, a complete left adrenalectomy and partial excision of the lesion in the para-aortic/left para-iliac regions were performed.

Following the surgery, the patient's bleeding was controlled, and she was transferred to the Intensive Care Unit (ICU) for extensive postoperative care and observation. In the ICU, the patient's condition remained stable, with all vital signs within expected ranges.

The pathology report detailed the examination of two separate specimens. The first, sourced from the gallbladder, displayed features consistent with emphysematous cholecystitis. The second specimen, obtained from retroperitoneal lesions, was composed of multiple fragments of predominantly yellow fatty tissue, interspersed with patches of gray and orange, measuring a total of 12 × 7.5 × 3.5 cm. Histological analysis revealed a largely uniform yellow adipose tissue with sporadic gray and orange inclusions. The pathological conclusion for this specimen indicated involvement of adrenal and retroperitoneum, aligning with characteristics typical of a myelolipoma.

In the postoperative period, the patient exhibited clinical signs of sinus tachycardia, shortness of breath, and diminished consciousness the morning following the surgery. An urgent consultation with anesthesiology resulted in endotracheal intubation for airway management. Concurrently, a consultation with internal medicine was sought to evaluate the likelihood of pulmonary thromboembolism, which prompted the initiation of anticoagulant therapy with heparin. Subsequently, the patient's condition deteriorated, manifesting as ventricular tachycardia, necessitating cardiopulmonary resuscitation (CPR). Despite achieving temporary return of spontaneous circulation through defibrillation, the patient failed to respond to continued resuscitative measures and was declared deceased after 45 min of sustained CPR efforts.

## 3 Discussion

Instances of concurrent adrenal and extra-adrenal myelolipomas are rarely documented in the literature, making each new case report a valuable contribution to understanding the complexities of their management and the diagnostic challenges often encountered.

In our case, we report a 65-year-old woman with a history of Steven-Johnson syndrome, major depressive disorder, and hypothyroidism, who presented with epigastric pain. Alongside her complex medical history, she was diagnosed with emphysematous cholecystitis. However, the diagnostic journey took an unexpected turn when imaging revealed two unforeseen lesions near her left kidney. This finding challenged the initial singular focus on cholecystitis and underscored the importance of a comprehensive evaluation in atypical presentations. The complexities of managing her case were further highlighted by the complications encountered during surgical intervention, which unfortunately led to her postoperative demise. This case illustrates the unpredictable nature of managing intricate medical histories and emphasizes the need for detailed preoperative assessment and cautious surgical decision-making.

Kamran et al. [9] described a similar case of a 65-year-old man with an adrenal fossa mass, initially identified on abdominopelvic CT as a bi-lobulated, fat-containing lesion suggestive of myelolipoma. This patient underwent laparoscopic-assisted adrenalectomy, during which an unexpected second mass in the retroperitoneal area was discovered and excised. Postoperatively, both masses were diagnosed as myelolipomas, and the patient remained symptom-free for nine months.

In line with our findings, incidental detection of myelolipomas has been noted in other cases. For instance, Zieker et al. [10] reported a 75-year-old man with abdominal pain, mild diarrhea, and weight loss, who underwent abdominopelvic CT. The imaging revealed two fat-containing retroperitoneal masses initially suspected to be liposarcomas. Elevated CA 19–9 levels were noted, but other tests were normal. Surgical excision of both masses led to the diagnosis of concurrent extra-adrenal and adrenal myelolipomas, with the patient remaining symptom-free post-surgery.

Hamidi et al. [11] found that most myelolipomas are discovered incidentally, as in our case, with a small percentage presenting symptoms due to mass effect. Additionally, a literature review spanning several decades highlighted that abdominal discomfort and pain are the most common presenting symptoms in symptomatic cases [12].

The differential diagnosis for fat-containing retroperitoneal masses is extensive, including various tumors of different origins. While retroperitoneal liposarcoma is rare in the adrenal gland [13], it is a common fat-containing tumor in the retro peritoneum [14]. Our case initially raised a suspicion of liposarcoma, thus highlighting the critical importance of careful evaluation in cases of retroperitoneal myelolipomas.

## 4 Conclusion

In conclusion, this case illustrates the diagnostic and management challenges in patients with complex medical histories. The unexpected discovery of abdominal lesions in a seemingly straightforward case highlights the need for thorough diagnostics and cautious surgical planning. This report underscores the importance of adaptability in clinical decision-making and contributes valuable insights into the unpredictable nature of medical practice.

## Ethical approval

This study is not required for ethical approval in our institution because this is anonymous and does not contain any personal information.

## Sources of funding

No funding.

## Author contributions

Conceptualization (G.M.); Data curation (G.M. and D.Z.); Supervision (G.M.); Validation (G.M.); Visualization; Writing - original draft (D.Z., and M.I.); and Writing - review & editing (D.Z., and M.I.).

## Guarantor

Golnaz Moradi.

## Registration of research studies

None.

## Consent

Written informed consent was obtained from the next of kin of the patient for publication and any accompanying images. A copy of the written consent is available for review by the Editor-in-Chief of this journal on request.

## Declaration of competing interest

The authors declare that they have no conflict of interest.
